# Awareness of Varicose Veins Associated With the Use of Contraceptive Pills Among Women in Jazan, Saudi Arabia: A Cross-Sectional Study

**DOI:** 10.7759/cureus.76912

**Published:** 2025-01-04

**Authors:** Wedad Mawkili, Seham Zakri, Mona Hattan, Somaya Abass, Shatha Khalaf, Lujin Zakri

**Affiliations:** 1 Department of Pharmacology and Toxicology, Jazan University, Jazan, SAU; 2 College of Pharmacy, Jazan University, Jazan, SAU

**Keywords:** awareness, contraceptive, jazan, saudi arabia, varicose veins

## Abstract

Background

Varicose veins are a rare side effect of birth control pills, linked to hormonal changes that may weaken vein walls and valves, impair blood flow, and increase venous pressure. Factors like sedentary behavior, prolonged standing or sitting, and genetic predisposition may worsen the risk. Additionally, contraceptive hormones can promote fluid retention and venous stasis, further contributing to vein dilation.

The direct link between birth control pills and varicose veins remains unclear, as factors such as BMI, age, and pre-existing conditions also play a role. However, limited research exists, particularly in our region, highlighting the need for awareness and studies to address these potential risks and improve women’s health outcomes.

Aim

The current study aims to assess the women's awareness of varicose veins caused by the contraceptive pill in Jazan, Saudi Arabia. By assessing women’s understanding of this topic, the study seeks to uncover any prevalent misunderstandings, such as underestimating the role of hormonal changes or overlooking contributing lifestyle factors. This information will help guide awareness campaigns and educational efforts to ensure accurate and accessible information is provided, addressing the unique health concerns and knowledge gaps within our community.

Methods

This study uses the convenience sampling method and data was collected using a self-report online questionnaire targeting women in Jazan who have used contraception pills or who have been diagnosed with varicose veins (N=494). This is a cross-sectional survey with a target group of Saudi women aged 18 to 64 years old who live in Jazan, Saudi Arabia. Participants were recruited through online platforms, including social media channels commonly accessed by women in the community, and through healthcare facilities where contraceptive counseling is provided. This multi-platform approach aimed to capture a diverse range of participants, although the reliance on convenience sampling may still lead to the underrepresentation of certain groups.

Results

Seventy-six percent of participants were aware of varicose veins, with a mean score of 1.76 and a standard deviation (SD) of 0.421. This indicates a generally low level of awareness among participants. Sixty-eight percent of participants were aware of the connection between contraceptive pills and the likelihood of developing varicose veins while 31.8% were not.

Conclusion

The majority of participants were aware of oral contraceptives and side effects. Regarding varicose veins, while most participants recognized the condition, only 38.1% understood its risk factors. Additionally, 64% were uncertain about the link between contraceptive pill use and varicose veins, with just 7.5% fully attributing the condition to contraceptive use. The study also found that older women were more likely to associate contraceptive pills with varicose veins. In light of the findings, this study suggests that healthcare providers enhance health education efforts to raise awareness of the risk factors associated with varicose veins, particularly for women using contraceptive pills. For instance, public campaigns can use social media, community health centers, and educational materials to disseminate information about varicose veins, their risk factors, and the potential influence of contraceptive pills.

## Introduction

Varicose veins, a common symptom of chronic venous insufficiency, affect people of all ages, with prevalence ranging from 10% to 30% in males and 25% to 55% in females [1,2]. The condition becomes more common with age [3], making prevention essential, especially in aging populations. Risk factors include female gender, family history, obesity, hormonal drugs such as contraceptives, and lifestyle factors like prolonged sitting or standing [4-6].

Contraceptive methods, including pills, implants, and intrauterine devices, are widely used by women. However, these can increase vascular risks, including varicose veins, by affecting venous blood flow [7]. Hormonal contraceptives containing progestin and estrogen, which prevent ovulation, have been linked to venous issues such as varicose veins and deep vein thrombosis [7]. Pregnant women are also at higher risk due to hormonal changes and increased venous pressure [3].

Varicose veins are influenced by various factors, including gender, age, obesity, pregnancy, family history, and lifestyle. However, the specific mechanisms by which contraceptives contribute to varicose vein development are under-explored. Most studies focus on deep vein thrombosis rather than superficial venous insufficiency. Limited research exists on the comparative risks of different contraceptive methods (e.g., pills vs. intrauterine devices) in the context of varicose veins. Moreover, only a few studies have examined the interaction between lifestyle factors and contraceptive use in increasing vascular risks. A cross-sectional study in Riyadh reported a high prevalence of varicose veins (47.6%) among 380 female participants, identifying factors such as increased age, positive family history, high body mass index (BMI), educational level, number of pregnancies, and use of oral contraceptives as significant contributors [5]. Another study focusing on female schoolteachers in Saudi Arabia found that 20.7% were diagnosed with varicose veins, with prolonged standing (89.2%) and obesity (71%) being the most commonly reported risk factors [8]. There is debate about whether taking birth control pills can also lead to varicose vein development. It is crucial for women to be educated about these risk factors, especially in Saudi Arabia due to the lack of studies in this field. The current study aims to assess women's awareness of varicose veins caused by contraceptive pills in Jazan, Saudi Arabia. It is essential to address these factors to prevent the development of varicose veins [9-11]. Contraceptive pills, commonly used by women for birth control, have been extensively studied for their safety and side effects. While they are highly effective in preventing pregnancy, oral contraceptives have been associated with a range of side effects, including an increased risk of cardiovascular diseases, blood clots, and varicose veins [12]. Moreover, bilateral involvement is typically observed in the majority of cases (75-76%), whereas unilateral involvement is detected with equal frequency in both legs [13].

Globally, varicose veins affect 10-30% of adults, with women being more susceptible due to hormonal influences [1,12]. Studies, such as the Edinburgh Vein Study, report prevalence rates of 40% in women and 32% in men, with age being a key factor [14,15].

Despite evidence of a hereditary component, most data rely on familial histories [16]. Pregnancy and menopause, along with hormonal medications, exacerbate varicose vein risk due to changes in hormone levels, increased blood volume, and venous pressure [1,3,12]. Hormonal contraceptives, particularly those containing estrogen, reduce venous tone and increase venous compliance, contributing to venous insufficiency and varicose vein development [17,18].

There have been some studies that have investigated the connection between pregnancy and varicose veins. However, the majority of these studies did not take into account the potentially confounding effect of age, which may have contributed to some degree of bias in the findings [19]. Research on the role of hormonal contraceptives in varicose vein development is limited, with few studies addressing this association [20]. Similarly, the connection between hormone replacement therapy (HRT) and varicose veins remains underexplored [21].

To our knowledge, in Saudi Arabia, there is limited research on women’s awareness of varicose veins linked to contraceptive use. This study aims to fill that gap, emphasizing the importance of education and preventive measures to mitigate the risk of varicose veins in this population.

## Materials and methods

Study objectives

The specific objectives of this study are to assess and evaluate women's knowledge of varicose veins as a possible side effect of contraceptive pill usage, to identify factors that influence awareness levels of varicose veins among contraceptive pill users, and to explore the attitudes of women towards the risks associated with contraceptive pills in relation to varicose veins. To measure attitudes toward the risks associated with contraceptive pills and varicose veins, the study used a combination of self-reported questionnaires and Likert-scale assessments.

Hypothesis

The present study states the hypothesis that the awareness level of women in Jazan about the risk of varicose veins linked to contraceptive pill use is generally low to moderate, and women in Jazan perceive contraceptive pills as a potential risk factor for the development of varicose veins.

Research Questions

We will assess awareness using a structured questionnaire designed to evaluate participants’ knowledge about varicose veins and their potential link to contraceptive pills. The questionnaire will include factual questions about the condition and its risk factors. Perception will be evaluated using Likert-scale questions to capture participants’ beliefs and attitudes about the risk of developing varicose veins due to contraceptive pill use. This section will explore their subjective understanding and concerns about the relationship. The study addresses three primary research questions: First, how aware are women in Jazan of the potential for varicose veins to develop as a side effect of contraceptive pills? Second, what factors influence the level of awareness among women in Jazan regarding varicose veins caused by contraceptive pills? Third, how do women in Jazan perceive the connection between contraceptive pills and the development of varicose veins?

Methods

Study Design and Study Population

This is a cross-sectional survey with a target group of Saudi women aged 18 to 64 years old who live in Jazan, Saudi Arabia. The survey was conducted for a duration of three months between 01 January 2024 and 30 March 2024. Participants were recruited through online platforms, including social media channels commonly accessed by women in the community such as WhatsApp and Telegram, and through healthcare facilities where contraceptive counseling is provided. All the study participants completed a 22-question self‐administered questionnaire. Informed consent was followed to ensure compliance with the research.

Sample Size and Sampling Method

This study used a convenience sampling method. A total sample size of participants was calculated using the sample size calculator (calculator.net) (N=494) based on the number of women in Jazan, Saudi Arabia (545197) in the 18-65 age group, according to the CEIC Data in the Kingdom of Saudi Arabia (Jazan city) in 2023 [22].

Inclusion and exclusion criteria

The study includes women over the age of 18 who either currently use contraceptive pills, have been diagnosed with varicose veins, or are considering using contraceptive pills in the future. Additionally, participants must reside in Saudi Arabia to be eligible. The study excludes women younger than 18 and those residing outside of Saudi Arabia.

Data collection tool and plan

An online questionnaire was employed as the primary data collection tool, targeting a specific group of women. The questionnaire used in this study was pre-tested (piloted) on a small sample of women in Jazan to ensure its reliability and validity. Feedback from this pilot phase was used to refine the questions for clarity and appropriateness to the study’s objectives. While the survey contained 22 questions, efforts were made to ensure that the questions were concise and focused on the key issues, minimizing the risk of respondent fatigue. The questionnaire was structured into four sections.

The first section gathered demographic information from the participants, including their age, educational level, occupation/profession, and number of children.

The second section focused on assessing the participants' awareness of contraceptive pills. Questions in this section included whether they had ever heard of oral contraceptive pills, whether they had used them as a method of family planning, the duration of their use, and whether they were aware of non-oral contraceptive methods. Additionally, participants were asked if they knew the side effects of oral contraceptives, where they obtained information about contraceptives, whether they trusted the effectiveness of oral contraceptive pills, and if they believed that oral contraceptive pills affected their health, mood, or relationships.

The third section aimed to evaluate the participants' awareness of the symptoms of varicose veins. It included questions on whether they had heard about varicose veins, their knowledge of risk factors that increase the likelihood of developing varicose veins, and whether they followed any preventive measures to reduce this risk. Participants were also asked if they were aware that varicose veins could lead to serious complications, and if they could identify the symptoms of varicose veins.

The fourth section assessed the participants' awareness of varicose veins caused by contraceptive pills. This section asked whether they had heard of the potential link between varicose veins and the use of contraceptive pills, the source of their information, whether they believed that contraceptive pills were a primary cause of varicose veins, and if they were considering changing their method of family planning due to the perceived risk of varicose veins.

Data analysis

Descriptive statistics, including frequencies, percentages, means, and standard deviations, were used to summarize the demographic characteristics of the participants and their responses in each section of the questionnaire. Inferential statistical analysis was conducted to test the hypotheses. Chi-square tests were used to examine the relationship between demographic variables (e.g., age, education, number of children) and awareness of varicose veins and contraceptive pill use. The chi-square test helps determine whether the observed frequencies differ significantly from the expected frequencies if there are no associations between the variables. All statistical analyses were conducted using Statistical Package for the Social Sciences (IBM SPSS Statistics for Windows, IBM Corp., Version 26.0, Armonk, NY) software. A p-value of less than 0.05 was considered statistically significant for all tests, ensuring that the findings were robust and reliable. To handle missing or incomplete data, the study utilized listwise deletion, excluding incomplete responses from analysis. This approach ensures that each participant included in the analysis has complete data for the variables being examined. However, if a substantial amount of data were missing, multiple imputations could have been considered to estimate missing values based on other responses. This would help minimize bias and improve the reliability of the results. The results of the data analysis were presented in tables and figures.

## Results

Table 1 summarizes the demographic data of 494 participants in the study assessing women's awareness of varicose veins caused by contraceptive pills in Jazan, Saudi Arabia. Participants between 20 and 30 years were more familiar with contraceptives (59.1%) (n= 292), followed by participants between 30 and 40 years (20.9%) (n=103), and only 6.7% (n= 33) of participants aged between 18 and 20 were familiar with contraceptives. The majority (70.6%) (n=349) had university-level education, while 23.3% (n=115) had completed high school, and only 3.04% (n=15) had primary or secondary education. In terms of occupation, 50.4% (n=249) were unemployed, 25.5% (n=126) were employees, and 24.1% (n=119) were students. Regarding family size, 68.4% (n=338) of participants had fewer than three children, 21.3% (n=105) had three to five children, and 10.3% (n=51) had more than five children. This distribution could have implications for women’s contraceptive choices and awareness. Women with fewer children might be more likely to use oral contraceptives as a family planning method, which could contribute to their familiarity with contraceptive pills. Additionally, smaller family sizes might correlate with higher levels of education and awareness, as women with fewer children may have more resources and time to seek out information about contraceptive options and associated health risks.

**Table 1:** The demographic data of the participants

Category	Subcategory	Frequency	Percentage (%)
Age	18-20 years	33	6.68
	20-30 years	292	59.11
	30-40 years	103	20.85
	40-64	66	13.36
Education level	Primary education	15	3.04
	Secondary education	15	3.04
	High school	115	23.28
	University education	349	70.65
Occupation	Student	119	24.09
	Employee	126	25.51
	Unemployed	249	50.4
Number of children	Less than 3 children	338	68.42
	3-5 children	105	21.26
	More than 5 children	51	10.32

Women with larger families might have different perspectives on contraceptive use and varicose veins. They could potentially have more experience with various contraceptive methods, but also might be less likely to prioritize side effects due to a focus on family planning. This could suggest a gap in awareness of health risks associated with long-term contraceptive use, such as the development of varicose veins. Further analysis could reveal whether family size correlates with differing levels of awareness or attitudes toward contraceptive pills and their side effects.

Family size could also be influenced by socioeconomic status, which might affect awareness. Women with larger families may have different access to healthcare or educational resources than those with fewer children. Given that the study showed a high level of university education (70.6%), it may be inferred that women with fewer children tend to have better access to educational and healthcare information, potentially leading to higher awareness of the risks of contraceptive pill use. While the findings indicate a positive trend between higher education levels and better awareness of contraceptive risks, the occupation did not show a significant correlation. This suggests that formal education may play a more substantial role in informing women about health risks than occupational exposure to health information. Further statistical analysis, such as logistic regression, could provide more robust insights into how these variables interact.

In conclusion, the family size distribution in the study offers a potential avenue for understanding how personal and socioeconomic factors influence women’s knowledge and attitudes toward contraceptive use and varicose veins. Future research could explore these relationships in more depth, assessing whether family size is a significant predictor of awareness or use of contraceptive pills in this population.

Table 2 shows the overall awareness and perceptions regarding contraceptive pills among the study participants. The majority of participants (76.3%) (n=377) were familiar with oral contraceptives, though only 40.5% (n=200) used them for family planning. Among those who used birth control pills, 64.4% (n=318) had been using them for less than a year. For those not using the pills, the primary reasons were a preference for other contraceptive methods (40.9%) (n=202) and fear of side effects (37.7%) (n=186). A significant portion (81.2%) (n=401) were aware of the potential side effects, and 76.3% (n=377) knew of other non-oral contraceptive options. The primary sources of contraception information were medical websites (34.2%) (n=169) and doctors (32.4%) (n=160). Despite concerns, 54.7% (n= 270) of participants trusted the effectiveness of birth control pills. Interestingly, 91.1% (n=450) did not feel that using birth control pills affected their health, mood, or relationships. This data highlights the overall awareness, usage patterns, and perceptions regarding contraceptive pills among women in the study.

**Table 2:** Women's awareness of oral contraceptive pills Chi-square tests were employed. A p-value of <0.05 was considered statistically significant.

Category	Subcategory	Frequency (494)	Percentage (%)	Mean	SD
Familiarity with oral contraceptives	Yes	377	76.3	1.76	0.425
	No	117	23.7		
Using birth control pills as a means of family planning	Yes	200	40.5	1.40	0.491
	No	294	59.5		
Duration of birth control pill usage if currently being used	Less than 1 year	318	64.4	1.84	1.32
	1 to 3 years	48	9.7		
	3 to 5 years	63	12.8		
	More than 5	18	3.6		
	I do not use birth control pills	47	9.5		
Reasons for not using birth control pills (IN CASE not to use them)	Due to the health condition	59	11.9	2.79	1.10
	Fear of unpleasant side effects	186	37.7		
	Anxiety and fear of long-term use	47	9.5		
	Desire to use another method of contraception	202	40.9		
Knowledge of other non-oral contraceptive methods	Yes	377	76.3	1.76	0.425
	No	117	23.7		
Knowledge of the side effects of birth control pills	Yes	401	81.2	1.81	0.391
	No	93	18.8		
The source of the contraception information	Contact a doctor	160	32.4	2.48	1.16
	Mobile apps	50	10.1		
	Search medical websites	169	34.2		
	Other	115	23.3		
The trust the effectiveness of birth control pills	Yes	270	54.7	1.54	0.498
	No	224	45.3		
Feeling that birth control pills affect your health, mood or relationship	Yes	450	91.1	1.91	0.285
	No	44	8.9		

The gap between high awareness and low usage rates of oral contraceptives could suggest that while women are knowledgeable about various contraceptive methods, they may have concerns or reservations about using oral contraceptives. These concerns could stem from potential side effects, such as weight gain, mood changes, or the risk of varicose veins. Additionally, cultural factors or misconceptions surrounding hormonal contraceptives might contribute to reluctance. If women are more familiar with non-oral methods (e.g., intrauterine devices, implants, or barrier methods), they may perceive these options as safer or more effective, despite limited experience or understanding of their benefits and risks.

In conclusion, while awareness of non-oral contraceptives is high, the relatively low usage of oral contraceptives highlights the importance of addressing underlying concerns, providing accurate information, and ensuring that women have access to the method that best suits their health needs and lifestyles. Further research and education are needed to bridge the gap between awareness and use, fostering informed decision-making in contraceptive choices.

The majority of women (76.9%) (n=380) were aware of varicose veins, with a mean score of 1.76 and a standard deviation (SD) of 0.421. However, only 38.1% (n=188) were aware of the risk factors that increase the likelihood of developing varicose veins, with a mean score of 1.38 and an SD of 0.486. When asked if they take preventive measures, only 23.7% (n=117) reported doing so, with a mean score of 1.23 and an SD of 0.425. Nearly half (49.6%) (n=245) of the participants knew that varicose veins could lead to serious complications, such as phlebitis or blood clots, while 50.4% (n=249) were unaware of this. The expectation that varicose veins would worsen with age was high, with 69.4% (n=343) of women believing so. In terms of symptoms, 19% (n=94) of women identified leg pain as a symptom, while 30% (n=148) pointed to swelling of the veins under the skin, and 45.1% (n=223) recognized all the listed symptoms (including menstrual irregularities, weight gain, and depression/anxiety) as indicators of varicose veins. The mean score for symptom recognition was 3.71 with an SD of 2.16, indicating variability in symptom awareness among the respondents (Table 3).

**Table 3:** Women's awareness of varicose vein symptoms Chi-square tests were employed. A p-value of <0.05 was considered statistically significant.

Category	Subcategory	Frequency (494)	Percentage (%)	Mean	SD
Awareness of varicose veins	Yes (aware)	380	76.9	1.76	0.421
	No (not aware)	114	23.1		
Awareness of the risk factors that increase the likelihood of developing varicose veins	Yes (aware)	188	38.1	1.38	0.486
	No (not aware)	306	61.9		
Doing any preventive measures to reduce the risk of developing varicose veins	Yes	117	23.7	1.23	0.425
	No	377	76.3		
Knowing that varicose veins can cause serious complications such as phlebitis or blood clots	Yes	245	49.6	1.49	0.500
	No	249	50.4		
The expectation of whether varicose veins worsen with age	Yes	343	69.4	1.64	0.461
	No	151	30.6		
Which of the following symptoms are symptoms of varicose veins:	Leg pain	94	19	3.71	2.16
	Swelling of the veins under the skin	148	30		
	Menstrual irregularities	14	2.8		
	Weight gain	11	2.2		
	Depression/anxiety	4	0.8		
	All of the above	223	45.1		

Table 4 provides an overview of the awareness among women regarding the risk of varicose veins linked to contraceptive pill use. It shows that 68.2% (n=337) of women are aware of the connection, while 31.8% (n=157) are not. The primary sources of information were friends (26.7%) (n=132) and media/internet (20%) (n=99), with only 15.8% (n=78) receiving information from a doctor. When asked about the impact of birth control pills on varicose veins, 64% (n=316) of women indicated uncertainty, while 7.5% (n=37) believed that contraceptive pills fully cause varicose veins, and 16.2% (n=80) believed they partially cause the condition. In terms of changing their birth control method due to the perceived risk, 25.9% (n=128) of women were sure they would make a change, while 51.4% (n=254) reported not using birth control pills at all.

**Table 4:** Women's awareness of varicose veins caused by birth control pills Chi-square tests were employed. A p-value of <0.05 was considered statistically significant.

Category	Subcategory	Frequency (494)	Percentage (%)	Mean	SD
Awareness of a connection between birth control pills and varicose veins	Yes (aware)	157	31.8	1.31	0.466
	No (not aware)	337	68.2		
The source of information regarding varicose veins	Doctor	78	15.8	3.14	1.52
	Friend	132	26.7		
	Media/internet	99	20		
	Educational brochure/booklet	10	2		
	I have never heard of varicose veins	175	35.4		
Thinking that birth control pills are the main cause of varicose veins	Fully affects	37	7.5	3.32	0.997
	Partially affects	80	16.2		
	No, there are other factors.	61	12.3		
	I don't know	316	64		
Thinking of changing your birth control method because of the risk of varicose veins	Sure	128	25.9	3.38	1.77
	I'm not sure	78	15.8		
	I'm not worried	20	4		
	There is no risk	14	2.8		
	No use of birth control pills	254	51.4		

The reliance on non-professional sources like friends (26.7%) and media/internet (20%) for information, compared to only 15.8% of women receiving guidance from doctors, suggests significant gaps in medical advice. This highlights the potential risks of misinformation and the importance of healthcare professionals actively participating in education efforts. Relying on friends or the internet could contribute to misconceptions about contraceptive use and varicose veins, underscoring the need for reliable, doctor-led campaigns.

The 64% of women who indicated uncertainty about the impact of contraceptive pills on varicose veins is concerning and suggests that many may not have clear information about the risks. This uncertainty presents an opportunity to strengthen educational outreach and increase patient awareness, particularly by providing accurate information through trusted sources like healthcare professionals.

While 25.9% of women indicated they would consider changing their contraceptive method due to the perceived risk of varicose veins, it is important to understand the rationale behind this decision. Women may be influenced by fear of potential side effects or misinformation, which calls for evidence-based counseling on the risks and benefits of contraceptive methods.

The 51.4% of women who do not use birth control pills could suggest that these women are either already aware of the potential risks or prefer alternative contraceptive methods. This group could provide insights into the broader study population and their contraceptive preferences, helping to inform public health strategies.

The implications of these findings for public health and clinical practice include the need for more targeted education campaigns, particularly those led by healthcare professionals. By improving awareness and addressing misconceptions, healthcare providers can better support informed decision-making and potentially reduce the risk of varicose veins and other related complications.

The results of the chi-square test indicate a statistically significant association between age and women's perception that contraceptive pills are the main cause of varicose veins (p=0.012) (Table 5). This suggests that women’s views on the role of contraceptive pills in causing varicose veins vary significantly across different age groups.

**Table 5:** Summary of chi-square test for the association between the demographic data and perception of contraceptive pills as a cause of varicose veins *The p-value is less than 0.05.

Demographic Data	Pearson Chi-Square	Degrees of Freedom (df)	P-value
Age	21.136	9	0.012*
Education level	8.676	9	0.468
Occupation	6.005	6	0.423
The number of children	8.728	6	0.189

The results also suggest that education level, occupation, and number of children do not significantly influence women's perception of contraceptive pills as a cause of varicose veins. There is no statistically significant association between the variables.

## Discussion

This study aimed to assess the level of awareness among women in Jazan, Saudi Arabia, regarding the potential association between the use of contraceptive pills and the development of varicose veins. The findings show that the majority of participants (76.3%) were familiar with oral contraceptives, although only 40.5% used them for family planning purposes. This finding aligns with previous research suggesting that while awareness of contraceptive methods is generally high, actual usage remains lower due to a variety of factors, including personal preferences and concerns about side effects [23]. For participants not using birth control pills, the primary reasons were a preference for other contraceptive methods (40.9%) and fear of side effects (37.7%). These concerns are consistent with other research, which shows that fear of side effects, including weight gain, mood changes, and health risks like blood clots, can deter women from using oral contraceptives [24].

The study also found that 76.3% of participants were aware of other non-oral contraceptive options, and 81.2% were aware of the side effects of birth control pills. This high level of awareness is critical as it provides women with choices that best fit their health needs and personal preferences. Research supports the idea that expanding knowledge of diverse contraceptive methods increases the likelihood that individuals will find a method they are comfortable with, leading to more consistent and effective use of contraception [25].

Medical websites (34.2%) and doctors (32.4%) were identified as the primary sources of information about contraception. Despite the concerns about side effects, 54.7% of participants trusted the effectiveness of birth control pills. Trust in the efficacy of contraception is essential for its continued use. The results indicate that while most participants are familiar with varicose veins (76.9%), only a minority (38.1%) are aware of the risk factors that increase the likelihood of developing this condition. This is consistent with previous studies which have highlighted that knowledge of specific risk factors for venous disorders remains low among women, even when general awareness of varicose veins is high [26]. The lack of awareness about the risk factors can result in fewer women taking preventive measures, as evidenced by the finding that only 23.7% of the participants engage in any preventive behaviors to reduce the risk of developing varicose veins.

One of the most significant findings of this study is the varied perception of the role of contraceptive pills in causing varicose veins. Most of the participants (64%) indicated uncertainty regarding the relationship between contraceptive pills and varicose veins. This is similar to other studies, where women showed low awareness of the potential venous complications associated with oral contraceptive use [27]. Despite this uncertainty, 7.5% of women believe that contraceptive pills fully cause varicose veins, while 16.2% believe that they partially contribute to the condition.

Oral contraceptives are known to affect venous tone and blood flow, contributing to venous stasis and increasing the risk of venous disorders [27]. However, it is important to highlight that the risk of varicose veins from contraceptive pill use is influenced by additional factors, such as genetics, age, and pre-existing venous insufficiency. The Chi-square test results demonstrate a statistically significant association between age and women's perceptions of contraceptive pills as a cause of varicose veins (p=0.012). This suggests that older women may have different views or experiences regarding the use of contraceptive pills and their venous health, possibly due to increased awareness of age-related risks. However, the results also indicate that education level, occupation, and number of children do not significantly influence these perceptions. This is somewhat surprising, as previous studies have found that education level can play a significant role in health literacy, particularly regarding contraceptive use [23].

A study by Baghdadi et al. (2022) explored the prevalence of varicose veins and risk factors among nurses in Saudi Arabia and examined how educational background and occupation affected health-related knowledge. The study found that education level was a significant factor in understanding health risks and adopting preventive measures, such as reducing prolonged standing at work, which can exacerbate venous insufficiency [5]. This study supports the role of education in improving health literacy in the Saudi population.

Moreover, Pazol et al. (2015) conducted a study in the United States that demonstrated that education programs targeted at increasing contraceptive knowledge significantly improved decision-making regarding contraceptive methods. Participants with higher educational levels were more likely to report understanding the risks and benefits of different contraceptive options, including oral contraceptives [22]. This supports the notion that education enhances awareness and informed decision-making related to contraceptive use.

Nonetheless, a study conducted in San Diego by Criqui et al. (2003) assessed the prevalence of chronic venous disease (including varicose veins) among diverse populations and found that education level was positively associated with awareness of venous health risks [12]. Participants with higher education were more likely to recognize the connection between lifestyle factors (such as contraceptive use) and venous disorders. However, cultural factors also influenced perceptions and behaviors, which may explain the differences in findings across various demographic groups.

The study also found that women rely on various sources of information for their knowledge of contraception and venous health, with the internet and doctors being the primary sources. This highlights the role of accessible health information in shaping women's perceptions of birth control risks. Moreover, despite concerns about varicose veins, 54.7% of participants expressed trust in the effectiveness of birth control pills, demonstrating a complex balance between health concerns and reliance on contraceptive efficacy. This aligns with findings from other research, where women acknowledged the risks associated with contraceptive use but prioritized effectiveness in preventing pregnancy [27-29].

Based on the findings, this study recommends that healthcare providers improve health education initiatives to increase knowledge of the risk factors linked to varicose veins, especially among women utilizing contraceptive pills. Public health campaigns ought to advocate for lifestyle adjustments, including regular physical exercise, weight management, and the avoidance of extended sitting or standing, to prevent venous diseases. Information sources must be diverse, including community outreach, social media, and instructional booklets to deliver accurate and thorough information. Additional investigation is required to examine the long-term implications of contraceptive pill usage on venous health, as well as the influence of genetic susceptibility and other risk factors.

Limitations

This study has several limitations, including its reliance on self-reported data, which may introduce recall bias. Furthermore, the cross-sectional design does not allow for an assessment of changes in awareness or perceptions over time. The study's sample size of 494 participants (approximately 0.09% of the total female population in Saudi Arabia) may limit the generalizability of the findings due to the small number.

## Conclusions

To conclude, the study found that a majority of women (76.3%) are aware of oral contraceptives and their side effects, but only 40.5% actively use them for family planning. The main reasons for not using oral contraceptives were concerns about side effects and a preference for alternative methods. Most participants were familiar with varicose veins, but only 38.1% knew about risk factors. The study also found a significant uncertainty about the relationship between contraceptive pill use and varicose vein risk, with only 7.5% attributing it to contraceptive use. Age was found to be a significant factor in women's perceptions of the relationship between contraceptive pills and varicose veins, suggesting older women may have heightened awareness.

The finding that age significantly influences women’s perceptions of the relationship between contraceptive pills and varicose veins is important because it highlights the need for age-specific education and interventions. Older women may have heightened awareness, potentially due to greater experience or concern about health risks. Understanding this can guide targeted education strategies, ensuring that younger women, who may be less aware, receive appropriate information about the potential risks of contraceptive pills. Tailoring interventions to different age groups could improve overall awareness and health-seeking behavior, ultimately promoting better decision-making regarding contraceptive use and venous health.

## Appendices

Survey

Assessment of Women’s Awareness of Varicose Veins Caused by the Contraceptive Pill in Jazan, Saudi Arabia Survey

This survey aims to assess measuring women's awareness of varicose veins caused by contraceptive pills and their impact on their health and quality of life.

Instructions: Please answer the following questions honestly and accurately. There are no right or wrong answers. Answers will be confidential and do not affect your rights or wrong answers. Answers will be confidential and do not affect your rights or interests.

**Table 6:** The demographic data

Section 1: The Demographic data	
1- Age	A. Less than 20 years old B. 20-30 years C. 30-40 years old D. Over the age of forty
2- Education level	A. Primary education B. Intermediate education C. High school education D. University education
3- The jop/profession	A. Student B. Employee C. Unemployed
4- Number of children	A. Less than 3 children B. 3 - 5 children C. More than 5 children

**Table 7:** The level of women's awareness of the types of contraceptive pills

Section 2: the level of women's awareness of the types of contraceptive pills	
1- Have you heard about oral contraceptives?	A. Yes B. No
2- Do you use birth control pills as a method of family planning?	A. Yes B. No
3- If you are using birth control pills, how long have you been using them?	A. less than one year 1 to 3 years B. 3 years to 5 years C. More than 5 years D. I do not use birth control pills
4- If you do not use birth control pills, what are the reasons that prevent you from using them?	A. Because of your health condition B. Fear of annoying side effects C. Anxiety and fear of the long duration of contraceptive use D. Desire to use another method of contraception
5- Do you know other non-oral contraceptive methods?	A. Yes B. No
6- Do you know the side effects of birth control pills?	A. Yes B. No
7- Where do you get contraceptive information from?	A. Communication with the doctor B. mobile applications C. Search medical sites on the Internet D. Other means
8- Do you trust the effectiveness of birth control pills?	A. Yes B. No
9- Do you feel that birth control pills affect your health, mood or relationship?	A. Yes B. No

**Table 8:** Women's level of awareness of symptoms of varicose veins

Section 3: Women's level of awareness of symptoms of varicose veins	
1- Have you heard about varicose veins?	A. Yes B. No
2- Do you know what are the risk factors that increase the possibility of developing varicose veins?	A. Yes B. No
3- Do you follow any preventive measures to reduce the risk of developing varicose veins?	A. Yes B. No
4- Did you know that varicose veins may cause serious complications such as inflammation of the veins or blood clots?	A. Yes B. No
5- Do you expect varicose veins to increase with age?	A. Yes B. No
6- Which of the following symptoms are symptoms of varicose veins:	A. Pain in the legs B. Swollen veins under the skin C. Disorder in the menstrual cycle D. overweight E. Depression anxiety F. all of the above

**Table 9:** Women's awareness of varicose veins caused by contraceptive pills

Section 4: Women's awareness of varicose veins caused by contraceptive pills	
1-. Have you heard about varicose veins caused by birth control pills?	A. Yes B. No
2- If you have heard about varicose veins, from what source did you hear about them?	A. Doctor/Medical B. Girlfriend/relative C. Media/Internet D. Brochure / educational booklet E. I have not heard of varicose veins
3- Do you think that contraceptive pills are the main cause of varicose veins?	A. fully affect B. Partially affected C. No other factors D. I don't know
4- Are you considering changing your family planning method due to the risk of varicose veins?	A. certainly B. I'm not sure C. I'm not worried D. There is no risk E. I do not use birth control pills
